# Biomedical imaging research: a fast-emerging area for interdisciplinary collaboration

**DOI:** 10.2349/biij.7.3.e21

**Published:** 2011-07-01

**Authors:** Z Sun, KH Ng, N Ramli

**Affiliations:** 1 Department of Imaging and Applied Physics, Curtin University of Technology, Perth, Australia; 2 University of Malaya Research Imaging Centre, Kuala Lumpur, Malaysia

## INTRODUCTION

Biomedical imaging has been undergoing rapid technological advancements over the last several decades and has seen the development of many new applications. Techniques such as functional imaging, spectroscopic imaging, optical imaging, and image-guided interventional techniques (treatment and therapy) have been gaining acceptance in areas ranging from basic research to clinical applications, and from the cellular level to the whole-organ level. Biomedical imaging is an interdisciplinary field that requires collaboration among biologists, chemists, medical physicists, pharmacologists, computer scientists, biomedical engineers, and clinicians of all specialties. Skills and tools provided by radiologists are at the centre of many clinical research programs [1]. Biomedical imaging is increasingly emerging as an essentially basic clinical investigational tool. Furthermore, imaging is becoming increasingly important as an approach to synthesise, extract and translate useful information from large multidimensional databases accumulated in research frontiers such as functional genomics, proteomics, and functional imaging [2].

## BIOMEDICAL IMAGING RESEARCH-FRONTIER AREAS

### Functional, metabolic and molecular imaging

Due to rapid technological developments leading to high spatial and temporal resolution, ultrasound, CT and MRI offer many opportunities for performing functional assessment of different body systems. The emergence and development of functional MR imaging and radionuclide imaging (SPECT/PET) have revolutionised the study of the functions of many body systems. Functional magnetic resonance (MR) encompasses a spectrum of techniques that depict physiological and molecular processes before morphological changes are visible on conventional imaging. Intraoperative MR guidance for neurosurgery improves prevision of tumour resection with use of combined data between functional MRI and SPECT [3].

MR spectroscopy is considered to be a method of molecular imaging. The ability to break down the chemical composition of a lesion has contributed significant information to the understanding of the metabolic process of normal and pathological tissues. The techniques most often used in clinical practice are proton spectroscopy (^1^HMRS) [4, 5] and phosphor spectroscopy (^31^PMRS) [6]. The former techniques give various information about acethylaspartate acid-neuronal cell health marker, creatinin-related to metabolic change, choline-element of cell membrane turnover, lactates-marker of anaerobic metabolism, and lipid-marker of mobile lipids. The latter technique offers evaluation of energetic condition of the cells, phosphate compounds, mono-biphosphate, phosphocreatinin, and adenosine triphosphate ATP [7]. With rapid advances in technology over the last decade, MR spectroscopy has entered the clinical routine [8, 9], and is now routinely used as an adjunct method to MRI for the pre-therapeutic diagnosis, assessment of therapy response, and therapeutic monitoring of brain, breast, and prostate cancer [10–14]. The relative concentrations of key chemical constituents such as citrate, choline and creatinine can be displayed on MR spectroscopy examination. In addition, MR spectroscopy has been used to help in the estimation of tumour volume, extracapsular extension and post-radiotherapy recurrence [15, 16].

PET and SPECT are powerful techniques capable of imaging biochemical processes *in vivo* in real-time. The use of PET and SPECT lies in monitoring the presence and activity of disease as a function of treatment, particularly in oncology. New functional and metabolic imaging techniques, especially hybrid SPECT/CT, PET/CT and PET/MRI, provide pathological information in addition to morphological information. Combined PET/CT offers superior advantages over each individual imaging modality and has been shown to be valuable in many applications such as cardiac imaging [17]. It has been reported that PET/CT allows more precise detection and localisation of coronary artery disease than individual PET or CT imaging modalities [18–20].

Molecular imaging is a diagnostic method based on the observation of cells and molecular structures *in vivo* using different imaging modalities and new diagnostic and therapeutic markers. Scintigraphy is in the forefront of molecular imaging because it can directly visualise molecular events occurring at subnanomolar to milimolar levels. Findings in PET and SPECT will continue to direct the progress of molecular imaging [21]. Molecular imaging contributes to biomedical research and development in many ways. In clinical practice, molecular imaging technologies may allow the detection of some disease processes years before they cause symptoms or before they would have been detected by means of conventional diagnostic tests or imaging modalities [22]. Molecular imaging enables the visualisation of molecular events and improves understanding of the drug actions within the body systems. Biomarkers at the molecular level could allow physicians to tailor therapy to the unique molecular profile of a patient or a disease, such as cancer [23].

### Biomedical optical imaging

Optimal imaging techniques use light emitted through fluorescence or bioluminescence. Biomedical optical imaging is used to investigate tissues from the organelle level to the organ level, to assist in the detection, diagnosis, and treatment of pathological processes in noninvasive or minimally invasive ways to the body. Applications of biomedical optical imaging range from the use of fluorescent and bioluminescent techniques to help identify biomolecular distributions within cells, to micrometre-scale cross-sectional imaging of the retina, to imaging and selective treatment of tumours [24, 25]. Because of the limited penetration of light in tissue, the large amount of photon scattering, and the need to transfect cells, neither bioluminescence nor fluorescence imaging is likely to become widely accepted.

New optical imaging techniques include optical coherence tomography, multiphoton microscopy, total internal reflection fluorescence, and speckle microscopy. Research priorities that are currently being performed or will be targeted include: *in vivo* imaging at cellular level, to evaluate early neoplastic changes, to enhance molecular imaging performance, to develop novel imaging devices such as image-guided biopsy probes or devices for imaging inside the body, and portable low-cost optical imaging methods for brain function (3).

### Computerised analysis of medical images-computer aided detection and diagnosis

The classic computer applications are computer-aided detection and computer-aided diagnosis (CAD) in mammography, lung and colon cancer [26–33].

Screening mammography was the first modality to benefit from CAD and clinical studies of CAD system have been conducted successfully to demonstrate the additional value it offers for the detection of breast cancer. The currently accepted model for CAD in mammography is to use it as a “second reader”—that is, the radiologist first searches the images for masses and microcalcifications, and then the computer assists the radiologist to identify additional suspicious regions that may be cancerous, with the final diagnosis and decisions concerning patient care being made by the radiologist [26, 27]. Use of CAD in the diagnostic work-up may prevent a malignant lesion from being misclassified as benign by the radiologist. It has also been reported that CAD can even improve cancer detection over double-read mammograms [28]. A recent systematic review of CAD in cancer imaging diagnosis reported that the use of CAD significantly improved the mean sensitivity, specificity and diagnostic odds ratio of cancer diagnosis using mammography and breast ultrasound compared with radiologists alone using the mammography and breast ultrasound methods [34].

The early detection and removal of colonic polyps can substantially reduce the risk of developing colon cancer. CT colonography, or virtual colonoscopy, has shown promise as a minimally invasive technique to help detect polyps [29, 30]. Manual reading of CT colonography images is increasingly prone to errors due to the high number of images to be analysed, which may lead to reader’s fatigue [35]. Moreover, image interpretation is subjected to reader’s bias and no systematic method has been designed so far for lesion visualisation [36]. Furthermore, there is a steep learning curve for the reader to acquire competence with image interpretation. CAD fulfills the role as a second reader and it has been shown to achieve high sensitivity for detecting colonic polyps. CAD improves radiologists’ accuracy in detecting polyps and classifying the feature of polyps, by acting as a second reader to enhance the radiologists’ performance in interpreting CT virtual colonoscopic examinations, especially for less-experienced readers [29–31, 37].

The detection of subtle focal pulmonary opacities on chest radiograph and CT remains a challenge. While screening and related studies have shown that CT is very sensitive for the detection of lung nodules, it is not very specific for the detection of lung cancers. Hence, there is substantial interest in the development of CAD systems to assist radiologists with the detection of nodules as well as characterisation of detected nodules as benign or malignant. CAD techniques are developed to improve interpretation in chest imaging with the aim of decreasing the intrinsic limitations and variations of human perception by alerting the reader to suspicious areas in chest radiographs and CTs [32, 33].

Studies have shown that CAD systems have improved the sensitivity in the detection of pulmonary nodules, especially for nodules smaller than 5 mm on CT examinations, which are often overlooked by visual inspection alone [32, 33]. The CAD technique can be used as a complementary tool that draws radiologists’ attention to certain image areas that need further evaluation. However, according to a recent systematic review of the effectiveness of CAD in cancer imaging, Eadie *et al*. found that using CAD to detect lesions on images provided less added value to radiologists than CAD diagnosis [34]. Thus, it seems likely that the newer generation of commercial CAD systems will be CAD diagnosis-based, which indicates the beneficial effects of CAD to radiologists by advising the status of the abnormality.

### Image-guided intervention and therapy

Biomedical imaging has played an important role in identifying and monitoring the effectiveness of the best treatments for many diseases. The ultimate goal of treatment is to identify the target of treatment and to deliver the maximum therapy to that target with minimal or no damage to the normal cells. Image-guided surgery improves the accuracy of many surgical procedures by eliminating much of the speculation, reducing the risk of human error, and minimising variations among the abilities of individual physicians [38, 39]. Image-guided therapy for cancer has improved local treatment and reduced the complication rate. With the advent and increasing application of robotics in conventional surgery, image-based navigational techniques are becoming more important and are expected to provide even better surgical outcomes in patients [40].

Real-time imaging guidance by means of CT, ultrasound or MRI is performed for various treatment options such as minimally invasive image-guided thermal ablation therapies for tumours of the liver, kidney, lung and bone (3). Image-guided minimally invasive therapies of the future may include the delivery of other therapeutics, including proteins, stem cells, gene therapies, chemotherapeutic agents and antibodies (3).

## BIOMEDICAL IMAGING AND INTERVENTION JOURNAL OFFERS AN OPPORTUNITY FOR INTEGRATION OF RESEARCHERS FROM DIFFERENT DISCIPLINES

The Biomedical Imaging and Intervention Journal (**biij**, available at http://www.biij.org) is an open access multidisciplinary peer-reviewed quarterly online journal, published by the Department of Biomedical Imaging, University of Malaya, Malaysia. **biij** is a multidisciplinary journal covering all the clinical and technical aspects of biomedical imaging and intervention, radiotherapy and oncology, minimally invasive image-guided therapy, image processing and informatics, molecular medicine, medical physics and radiobiology as well as radiography and bioengineering related to imaging or intervention. Researchers are encouraged to fully utilise the biij as a platform for biomedical imaging research by publishing their original research works and sharing research outcomes with colleagues from the Asia Pacific region and the rest of the world.
